# The Kinetics of Glomerular Deposition of Nephritogenic IgA

**DOI:** 10.1371/journal.pone.0113005

**Published:** 2014-11-19

**Authors:** Kenji Yamaji, Yusuke Suzuki, Hitoshi Suzuki, Kenji Satake, Satoshi Horikoshi, Jan Novak, Yasuhiko Tomino

**Affiliations:** 1 Division of Nephrology, Department of Internal Medicine, Juntendo University School of Medicine, Tokyo, Japan; 2 Department of Microbiology, University of Alabama at Birmingham, Birmingham, Alabama, United States of America; Radboud university medical center, Netherlands

## Abstract

Whether IgA nephropathy is attributable to mesangial IgA is unclear as there is no correlation between intensity of deposits and extent of glomerular injury and no clear mechanism explaining how these mesangial deposits induce hematuria and subsequent proteinuria. This hinders the development of a specific therapy. Thus, precise events during deposition still remain clinical challenge to clarify. Since no study assessed induction of IgA nephropathy by nephritogenic IgA, we analyzed sequential events involving nephritogenic IgA from IgA nephropathy-prone mice by real-time imaging systems. Immunofluorescence and electron microscopy showed that serum IgA from susceptible mice had strong affinity to mesangial, subepithelial, and subendothelial lesions, with effacement/actin aggregation in podocytes and arcade formation in endothelial cells. The deposits disappeared 24-h after single IgA injection. The data were supported by a fluorescence molecular tomography system and real-time and 3D in vivo imaging. In vivo imaging showed that IgA from the susceptible mice began depositing along the glomerular capillary from 1 min and accumulated until 2-h on the first stick in a focal and segmental manner. The findings indicate that glomerular IgA depositions in IgAN may be expressed under the balance between deposition and clearance. Since nephritogenic IgA showed mesangial as well as focal and segmental deposition along the capillary with acute cellular activation, all glomerular cellular elements are a plausible target for injury such as hematuria.

## Introduction

Immunohistological analysis is the crux of the diagnosis of IgA nephropathy (IgAN) for which dominant or co-dominant mesangial deposition of IgA is essential. Significant number of patients is reported to have elevated levels of circulating IgA immune complexes (IC), presumably due to aberrantly glycosylated IgA and endogenous antiglycan antibodies [1]–[3]. Thus, primary IgAN is an IC-mediated glomerulonephritis that is immunohistologically defined by the presence of glomerular IgA deposits accompanied by various histopathologic lesions. However, whether IgAN is attributable to mesangial IgA or IgA IC deposition remains unclear. One mystery is the lack of correlation between the intensity of deposits and the extent of glomerular injury; in addition, a long-standing question remains whether these mesangial IgA deposits directly induce hematuria [3]–[6]. To the best of our knowledge, no definitive study provides a comprehensive answer to these questions.

In contrast, we also know patients who exhibit minor clinical symptoms, such as very low levels of hematuria and proteinuria but have massive glomerular IgA depositions, suggesting that not all IgA immune deposits possess equivalent pathogenic potential. These conflicting clinical findings make it difficult to identify the true therapeutic target of this disease, thereby hindering the development of a specific therapy [7], [8]. Thus, despite almost half a century of clinical and basic research, the immunologic processes that induce and perpetuate glomerular IgA deposition and the detailed mechanism underlying deposition events remain unknown. IgAN may represent multiple diseases with abnormal urinary findings, sharing common pathogenic markers, such as mesangial IgA immune deposits, and having unknown mechanisms whereby damage is propagated in a discontinuous pattern [5]. The deposition of circulating IgA/IgA-containing IC may be the most likely mechanism; an understanding of the detailed sequential events in pathogenesis is the key to developing a successful therapy [2], [3], [9], [10].

We recently established a strain of spontaneous IgAN-prone gddY mice in which the disease phenotype and genetic regulation largely overlap with human IgAN [11]–[13]. Although abnormal glycosylation of *O*-linked glycans in the hinge region of human IgA1 is linked to human IgAN [2], [3], it has long been believed that *O*-glycans are absent in the hinge region of murine IgA. However, our recent paper [12] revealed that more severe disease in gddY mice occurs in those with an IgA allotype that has less sugar content in the hinge region. Thus, modification of carbohydrate structures may change the conformational or biochemical properties of murine IgA, thereby altering the nephritogenicity or formation of IgA-IC, as seen in human IgAN [2]. Moreover, aberrantly glycosylated IgA may induce macromolecular IgA complex formation, leading to the complement activation and subsequent progression to IgAN [14], as seen in human IgAN [2], [9]. Furthermore, serum levels of IgA-IgG IC correlate with the severity of renal damage in IgAN-prone mice [15]. Our recent reports demonstrate that cells responsible for producing nephritogenic IgA are disseminated in the mucosa-bone marrow axis [15]–[18] and are regulated by the innate immune system [19]–[21], as seen in human IgAN [13], [19], [22], [23]. Accordingly, experimental, immunological, and histopathological findings in this susceptible strain seem to recapitulate the clinical features of human IgAN [11]–[13]. Details of the sequence of events in glomerular deposition of nephritogenic IgA from IgAN have not been described in the literature. In the present study, we use nephritogenic IgA from this susceptible mouse strain to examine this sequence of events using a real-time imaging system. Sequential analysis of IgA-induced IgAN may provide clues to the pathogenesis of this disease and strategies for its treatment.

## Materials and Methods

### Mice

The gddY mice were established by the selective mating of early-onset ddY mice for more than 20 generations and have a 100 % incidence of severe disease, even at a young age [11]–[16]. The gddY mice were maintained on a regular chow (MF; Oriental Yeast, Tokyo, Japan) and water *ad libitum* in a specific pathogen-free (SPF) room at the animal facility of Juntendo University, Tokyo, Japan.

Same-age female Balb/c (Balb/c) mice were used as controls. Female Balb/c AJcl-nu/nu (nude) mice were used for the injection of serum or IgA from the gddY mice with early-stage disease and from the Balb/c mice. Balb/c and nude mice were purchased from CLEA Japan Inc., Tokyo, Japan. The experimental protocol of the present study was approved by the Ethics Review Committee for Animal Experimentation of Juntendo University Faculty of Medicine.

### The serum single-injection model

Serum samples were obtained from gddY mice aged between 20 and 25 weeks who showed severe renal injury with glomerular IgA deposits. Serum from age-matched healthy Balb/c mice was also collected as the control serum. Serum levels of IgA were measured using a single radioimmunodiffusion assay (SRL, Tokyo, Japan). The obtained serum samples, including 2 mg IgA of gddY or Balb/c mice (300 µL, diluted with saline) were injected once into the tail vein of nude mice aged between 10 and 12 weeks. The nude mice were euthanized either 2 or 24 h after the injection of serum. Kidneys were collected from the nude mice after perfusion with normal saline solution. The serum samples and kidneys were stored at −80°C until use. Renal histological analysis was performed using electron and confocal microscopy.

Snap-frozen 3-µm-thick renal sections were used for immunofluorescence with a fluorescein isothiocyanate-conjugated goat antimouse IgA antibody (BD Biosciences, San Diego, CA, USA; Pharmingen, San Diego, CA, USA). For electron microscopy, the renal specimens were fixed in 2 % glutaraldehyde, washed in phosphate buffer, and postfixed in 1 % osmic acid. The specimens were washed, dehydrated, and embedded in Epon 812. The ultrathin sections were contrasted with uranyl acetate and lead citrate and then examined under an electron microscope (Hitachi 7100, Tokyo, Japan).

### Purification of IgA

Serum samples were obtained from the gddY and Balb/c mice aged between 20 and 25 weeks. Each serum sample was incubated with a rat antimouse IgA antibody with CNBr Sepharose overnight at 4°C. After washing with phosphate-buffered saline (PBS), IgA was eluted with 0.1 M glycine (pH 2.5) using a fraction collector. Molecular size of purified IgA obtained from ddY and Balb/c mice were analyzed by Western blot analysis. The purified IgA samples were used for fluorescence molecular tomography and confocal laser microscopy analyses.

### Kinetic studies using a fluorescence molecular tomography system

These studies were conducted on female nude mice (n = 3). The purified IgA samples obtained from gddY or Balb/c mice were labeled with Vivo Tag-S 750 (VisEn Medical Inc. Woburn, MA, USA). Purified IgA samples were dissolved at 2 mg/mL in PBS, and 0.5 mL of the solution was alkalized with 50 µL of 1 M sodium bicarbonate solution. The mixture was incubated with Vivo Tag-S 750 for 1 h at room temperature, with stirring. The labeled IgA was purified using resin column chromatography. Fluorescein-labeled IgA (0.5 mg/mL) was dissolved in 200 µL PBS and injected into nude mice aged between 10 and 12 weeks. After 2, 4, and 24 h, IgA signals were measured using a fluorescence molecular tomography system (VisEn Medical Inc. Woburn, MA, USA).

### Kinetic studies by confocal laser microscopy

Kinetic studies by confocal laser microscopy were performed in female nude mice (n = 3). Purified IgA obtained from gddY or Balb/c mice was labeled using an Alexa Fluor 633 protein labeling kit (Molecular probes, Inc., Eugene, OR, USA). Purified IgA samples were dissolved at 2 mg/mL in PBS, and 0.5 mL of the solution was alkalized with 50 µL of a 1 M sodium bicarbonate solution. The mixture was incubated with Alexa Fluor 633 dye at room temperature for 1 h, with stirring. The labeled IgA was purified using resin column chromatography. Before analysis, the nude mice were anesthetized with sodium pentobarbital. An incision of approximately 10 mm was made on the lower back, and a kidney was removed using small forceps. The surface of the lower pole of the kidney was sliced and placed on a microscope stage. Dextran (500 kDa) is not filtered and is a good marker for blood vessel wall integrity. First, fluorescein-labeled 500-kDa dextran (Sigma–Aldrich Inc., St. Louis, MO, USA) was injected into nude mice to confirm the staining of glomeruli. Following this, fluorescein-labeled IgA (0.5 mg/mL) was dissolved in 200 µL PBS and injected into mice. After 1 and 120 min, the kinetics of labeled IgA in glomeruli were analyzed using confocal laser microscopy (ZEISS, LSM 510 META, Germany). Single or time-series images of glomeruli were recorded in fluorescence mode.

### The procedure of BMT

The procedure for murine BMT was previously described in detail [15]–[18]. In brief, BMC were harvested from the tibia, femur, and humerus of control Balb/c mice at 8–10 weeks of age under sterile conditions. Red blood cells were removed from the collected BMC. Grouped ddY mice develop glomerular lesions with mesangial IgA deposits within 6–8 weeks of age [11], [12]. Young and old gddY mice aged 8 and 20 weeks who were at the early stage of the disease were used as recipients. Following this, 10^7^ BMCs were injected into the tail vein of irradiated recipient mice (n = 3) at 700 rad. The recipient mice were housed under SPF conditions. Young and old gddY transplant recipients were euthanized 12 weeks after BMT. IgA deposits were assessed by immunofluorescence and electron microscopy, and urinary protein was measured by immunoassay (DCA 2000 system; Siemens Healthcare Diagnostics, Tokyo, Japan).

## Results

### A single injection of serum from gddY mice, but not from Balb/c mice, induced glomerular IgA deposition with an activation of glomerular podocytes and endothelial cells

**Figure 1:**
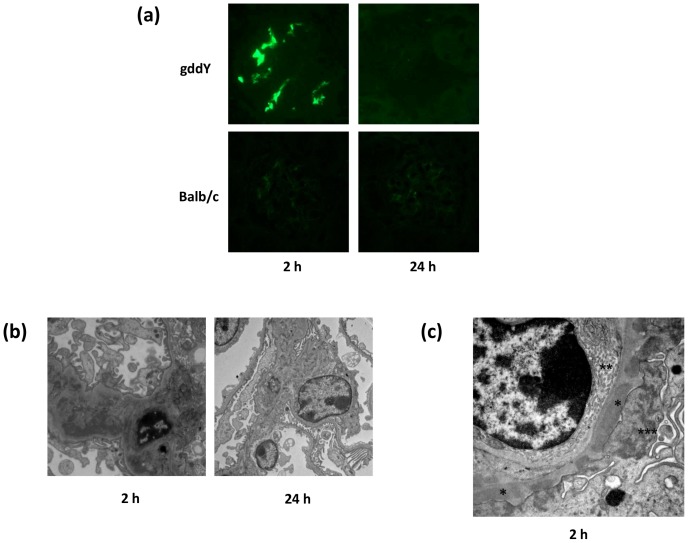
A single injection of serum from gddY mice induced glomerular IgA deposition with activation of glomerular podocytes and endothelial cells. (a) Glomerular IgA deposits were found at 2 h in mice injected with serum from gddY mice but not from Balb/c mice. These fluorescent signals disappeared after 24 h in this single-injection model. (b) These deposits and clearance were confirmed using electron microscopy. Electron-dense deposits were mainly detected in paramesangial lesions. (c) Some glomeruli showed subendothelial and subepithelial deposits (*) with arcade formation in glomerular endothelial cells (**) and effacement and actin aggregation in podocytes (***) 2 h after the injection.

We first tested whether a serum from gddY mice induced glomerular IgA deposition in nude mice. Serum from Balb/c mice was used as a control. Renal tissue was obtained from nude mice euthanized 2 and 24 h after a single injection of the serum. Serum from gddY mice, but not Balb/c mice, induced mesangial IgA deposition 2 h after the injection (Fig. 1a). The electron-dense deposits in the paramesangial areas were confirmed using electron microscopy (Fig. 1b). After 2 h, in some glomeruli of the mice injected with the gddY mouse serum, electron-dense deposits were detectable in subepithelial and subendothelial lesions, with effacement and actin aggregation in the podocytes and arcade formation in some endothelial cells. This data is indicative of the activation of those cells (Fig. 1c). Such morphological changes were not found with injections of Balb/c IgA (serum to be precise; data not shown). In this single-injection model, mesangial IgA deposition and electron-dense deposits almost disappeared after 24 h (Fig. 1a and b, right panels).

### IgA from gddY mice showed high affinity for kidney tissue

**Figure 2:**
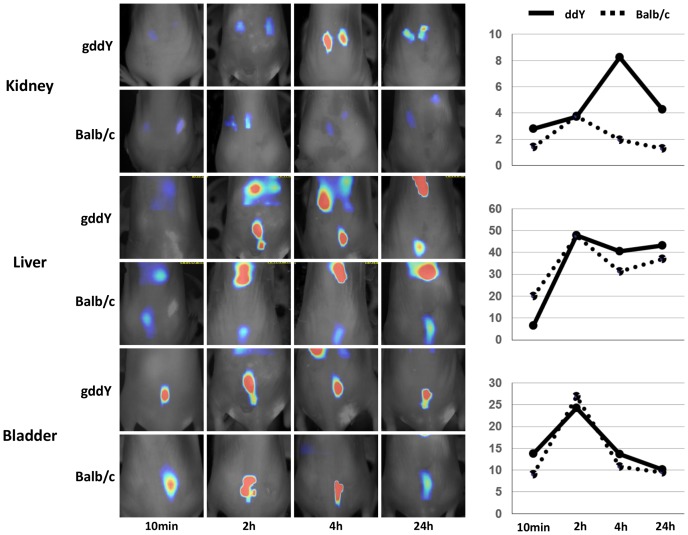
Kinetics of injected fluorescently labeled IgA in a fluorescence molecular tomography system. A fluorescence molecular tomography system (FMT) is capable of resolving size and concentration of fluorochromes in deep tissue *in vivo*. Fluorescein-labeled IgA samples from gddY and Balb/c mice were injected into nude mice and monitored from 10 min to 24 h postinjection by FMT. After 2 h, IgA signals in the liver and bladder were found in a similar manner in both the groups of nude mice. However, IgA signals in the kidneys clearly differed between them. Mice injected with gddY IgA showed strong signals in the kidneys, with a peak at 4 h.

We next labeled serum IgA from gddY mice and control Balb/c mice with Vivo Tag-S 750 and analyzed the *in vivo* kinetics of IgA using a fluorescence molecular tomography system after a single injection in nude mice. After the injection of IgA from gddY or Balb/c mice, fluorescence signals in the kidneys, liver, and bladder were evaluated after 10 min and 2, 4, and 24 h. There was no difference in the signals in the liver and bladder between the mouse groups that received labeled IgA from either gddY or Balb/c mice (Fig. 2, middle and lower panels). Two hours after injection, IgA signals were detected in the kidneys of both groups by molecular tomography (Fig. 2 upper panels). However, the renal signals in mice injected with IgA from gddY mice increased, with a peak at 4 h after the injection, whereas renal signals in controls were weak and gradually decreased.

### IgA from gddY mice is deposited along the glomerular capillary wall in a focal and segmental manner

**Figure 3:**
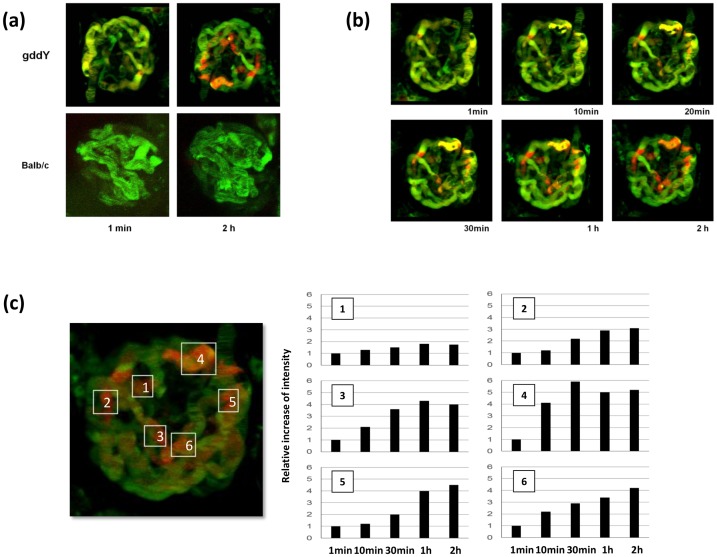
IgA from gddY mice is deposited along the glomerular capillary wall in a focal and segmental manner. Detailed kinetics of IgA deposition analyzed from 1 min to 2 h postinjection using confocal laser microscopy. Alexa Fluor 633-labeled IgA from gddY and Balb/c mice (red) and 500-kDa fluorescein-labeled dextran (green) were injected for analyzing kinetics of IgA deposition and visualizing blood vessel wall integrity, respectively. (a) IgA signals were detectable even after 1 min and accumulated up to 2 h in a focal and segmental manner in mice with IgA from gddY mice. In contrast, mice who received Balb/c IgA did not show a signal even after 2 h. (b)(c) Serial images of a glomerulus in mice with IgA from gddY mice showed that these IgA molecules accumulated on top of the initial aggregates along the glomerular capillaries. These aggregates were found in a focal and segmental manner but not in a diffuse and global manner.

To further clarify the sequential manner of the deposition process, we injected Alexa Fluor 633 protein-labeled IgA (red) from gddY and Balb/c mice and fluorescein-labeled 500-kDa Dextran (green) to visualize the glomerular capillary in the nude mice. We then analyzed the glomerular capillaries using confocal laser microscopy from 1 min to 2 h after the single injection (Fig. 3). Yellow images indicate IgA deposition along the glomerular capillaries. This *in vivo* real-time/3D imaging (Fig. 3a and supporting information in video and 3D images) confirmed the data from previous immunofluorescent analysis, electron microscopy, and tomography, in that IgA from gddY has a higher affinity for the glomerulus than that from Balb/c mice. Nevertheless, this gddY-derived IgA was deposited along the glomerular capillary in a focal and segmental manner. This *in vivo* imaging with a focus on the glomerular capillary showed initial deposition after only 1 min (Fig. 3b). IgA accumulated on top of these first aggregates or microdeposits (Fig. 3c); however, this accumulation was not diffuse. Two hours after the injection, the amount of labeled IgA in the glomerular capillary gradually decreased in both groups of mice. Nonetheless, a clear accumulation of IgA in glomerular mesangial areas was found in the *in vivo* imaging with a focus on mesangial areas after 2 h in the group injected with IgA from gddY mice, but not in that from Balb/c mice. Molecular forms of both IgA purified from ddY and Balb/c mice were analyzed by Western blotting under non-reducing condition. IgA purified from ddY mice serum was predominantly polymeric with a small peak of dimeric IgA. IgA purified from Balb/c mice was monomeric and dimeric dominant (data not shown).

### Glomerular IgA deposits in old gddY mice remained after bone marrow transplantation (BMT) despite improved proteinuria

**Figure 4:**
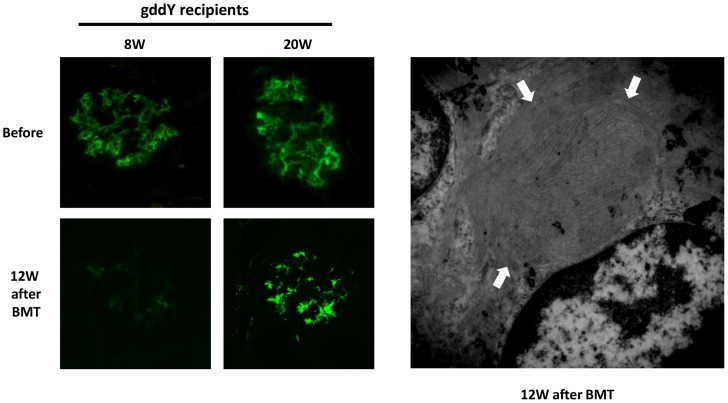
Glomerular IgA deposits in old gddY mice did not disappear after bone marrow transplantation (BMT) despite improvement in proteinuria. BM cells from healthy Balb/c mice were transplanted into young (8 weeks) and old (20 weeks) gddY mice at an early stage of disease. Although proteinuria was present in the young and old recipients 12 weeks after BMT, fluorescence analysis still detected glomerular IgA deposition in old gddY recipients but not in young gddY recipients (left panels). Electron microscopy still detected paramesangial dense deposits showing fibrous and lattice structures in the old recipients (right panel).

To clear IgAN in gddY mice [14], [15], we transplanted bone marrow cells (BMC) from Balb/c (healthy) mice into gddY mice. We confirmed the disappearance of proteinuria in both young and old recipients 12 weeks after BMT [albuminuria before vs. after BMT (µg/day): young recipients, 121.3±21.2 vs. 26.3±6.7; old recipients, 198.0±23.0 vs. 30.3±12.9]. However, fluorescence analysis still detected glomerular IgA deposition in old but not young gddY recipients (Fig. 4 left panels). Electron microscopy detected dense paramesangial deposits in the old recipients; however, these dense deposits showed fibrous and lattice structures.

## Discussion

The primary abnormal clinical manifestation of IgAN in patients is recurring bouts of hematuria with or without associated proteinuria [3], [4]. In the complex web of potential mechanisms implicated in the pathogenesis of IgAN, mesangial IgA deposits remain a *sine qua non*. This notion may lead us to believe that mesangial IgA deposition directly induces hematuria. However, there are no studies proving a direct relationship between the amount mesangial IgA immune deposits and the extent of glomerular injury leading to hematuria. Our earlier comprehensive observations emphasized the variable characteristics of the IgA/IgA IC, particularly the nature of antigens and the molecular form of IgA, as major determinants of glomerular injury [10]. Several physical characteristics, including size, lattice composition, electric charge, and glycosylation, may also influence the probability of deposition in the glomerular mesangium [2], [3], [9], [10], [24]–[31]. Sequential events were analyzed in the experiments with artificially modified IgA or IgA IC. Even these observations failed to provide a sufficient explanation for the mechanisms leading to hematuria in IgAN. This may be partly due to the absence of experiments with IgA preparations that have been confirmed to induce a chronic progression of IgAN with mesangial cell proliferation and matrix expansion. Thus, this appears to be the first study to assess the kinetics of glomerular deposition of nephritogenic IgA in IgAN [11]–[16].

The present analysis using tomography showed a similar time course of liver signals peaking at 2 h in both the groups of mice, indicating that most of the injected IgA from the gddY and control mice was trapped in the liver in a similar fashion. The parts of injected IgA from both the groups of mice passed through into the bladder in a similar manner. Nevertheless, renal signals were conspicuously stronger in the group injected with IgA from gddY mice than in those injected with IgA from healthy mice. This finding indicates that some clones of IgA in gddY mice have a strong affinity for kidney tissues and may be nephritogenic. Our recent studies have shown that IgA in gddY mice is polymeric and that it has a high capacity for activating complement cascades, including the lectin pathway [14]. The renal prognosis of this susceptible model is likely to be dependent on the content of carbohydrates in the hinge region of IgA [12]. Recent research has also suggested that aberrant N-glycan glycosylation may be involved in the pathogenesis of IgAN in mice [32], [33]. These findings suggest that aberrant modifications of serum IgA carbohydrate side chains are involved in the development of IgAN, not only in humans but also in mice, regardless of whether the carbohydrates are O-glycans or N-glycans [2], [3], [13]. Recent studies have revealed that aberrantly glycosylated IgA1 (GdIgA1) plays a nephritogenic role in this disease [1]–[3], [9], [34]. The serum level of GdIgA1 is associated with the disease activity of IgAN [35]. On the other hand, anomalous glycosylation of IgA is a key determinant of glomerular affinity [31]. Thus, the high glomerular affinity found in the present study may be partly due to the basic molecular nature of IgA, presumably with aberrant glycosylation.

The disappearance of the glomerular deposits in this single-injection model is one of most important findings of the present study. This finding indicates that a continuous clearance mechanism of IgA deposition may be at work in the glomerulus. Thus, glomerular IgA deposits in IgAN may be explained as an imbalance between deposition and clearance. Although most IgAN patients have undoubtedly had glomerulonephritis for years before a renal biopsy, it seems that a certain percentage of IgAN patients may go through a clinical period just after the onset, when neither IgA nor C3 is detectable in the renal biopsy but when there are histological lesions and aberrant urinary test results [7], [8]. Thus, our results indicate that the intensity of staining for IgA in the kidney may wax or wane based on the balance in the glomeruli or mesangium.

Increasing evidences suggest that mucosal type polymeric IgA1 is produced in BM of IgAN patients [36]. Furthermore, BMT in patients with leukemia and IgAN resulted in the cure of not only leukemia but also IgAN [37]. Therefore, cross talk between the mucosa and BM is suspected in the pathogenesis of IgAN [13], [38], [39]. We previously showed that murine IgAN can be reconstituted via BMT from ddY mice to healthy mice [15]–[18], [40]. We also showed that early-stage IgAN in ddY mice can be cured by removing glomerular IgA via BMT from healthy mice [15], [16]. However, glomerular IgA deposits in the present BMT model from healthy mice to ddY mice disappeared in young but not old ddY recipients; proteinuria in both young and old recipients was alleviated by BMT. This finding suggests that incompletely cleared IgA deposition in the disease course of gddY mice may change in conformation or biochemical properties as a result of their organization or fibrosis with mesangial matrix components, losing nephritogenicity. However, because these deposits were present in old ddY recipients after BMT, such organized IgA may still have epitopes that the fluorescence-labeled anti-IgA antibody recognizes. Therefore, present BMT models indicate that glomerular IgA in IgAN patients may be mixed with freshly-delivered nephritogenic IgA and non-inflammatory organized IgA, particularly in those with a longer disease history. This finding may also partly explain the discrepancy between the amount of IgA deposition and severity of glomerular lesions in human IgAN.

Although most glomeruli in mice injected with gddY IgA showed mesangial IgA deposition, deposits along capillaries were detected in a focal and segmental manner. *In vivo* serial imaging showed that such subendothelial/subepithelial IgA deposits seemed to be formed as a result of an accumulation on the focal/segmental initial aggregates of IgA, suggesting that the initial aggregates (microdeposits) may change the local physiological conditions, thereby leading to the increased glomerular affinity of IgA. These physiological changes may include the local deceleration of the glomerular capillary flow or increased permeability of the glomerular basement membrane interposed between the endothelial and epithelial layers [41]–[43]. These deposits along glomerular capillaries were indeed found along with morphological changes in glomerular endothelial cells and podocytes, even after 2 h. This phenomenon is suggestive of a rapid activation of glomerular resident cells by IgA deposition. We can speculate that such rapid activation (particularly in the endothelial cells, presumably in combination with slow blood flow) may facilitate leukocyte adhesion to the deposits and their subsequent clearance and/or inflammatory responses [44], [45], leading to hematuria in IgAN. This real-time imaging showed increased passage of IgA into glomerular mesangial lesions and the relevant glomerular pole after 2 h (data not shown), suggesting that subendothelial/subepithelial deposits may induce permeability factors, such as vascular endothelial growth factor (VEGF), and increase the flow of plasma into the mesangium and subsequent interstitium/lymph via the glomerular pole. Thus, nephritogenic IgA deposition may induce dynamic alterations in the glomerulus and subsequent glomerular and interstitial injury.

This study revealed the kinetics of glomerular deposition over the course of IgA-induced IgAN. These IgA molecules have a strong affinity for focal and segmental subendothelial, subepithelial, and glomerular mesangial lesions. Rapid cellular activation of endothelial cells and podocytes by IgA deposition may precede the events of hematuria in IgAN. The significant differences between human and murine IgAN limit the translation of these data to the human disease. Further, similar study using human GdIgA1 is needed. However, the present findings regarding the kinetics of IgA deposition may indicate that not only glomerular mesangial cells but also endothelial cells and podocytes are plausible targets of glomerular injury in IgAN.

## Supporting Information

Video S1 ***In vivo*** **3D imaging of glomerulus after injection of nephritogenic IgA.** 3D images of glomeruli at 2 hours after single injection of purified IgA from gddY mice were evaluated by a confocal laser microscopy. Alexa Fluor 633-labeled IgA from gddY (red) and 500-kDa fluorescein-labeled dextran (green) were injected for analyzing kinetics of IgA deposition and visualizing blood vessel wall integrity, respectively. IgA signals were detectable after 2 h in a focal and segmental manner in mice with IgA from gddY mice.
(MOV)

Video S2 ***In vivo*** **3D imaging of glomerulus after injection of IgA from Balb/c mice.** 3D images of glomeruli at 2 hours after single injection of purified IgA from Balb/c mice were evaluated by a confocal laser microscopy. Alexa Fluor 633-labeled IgA from and Balb/c mice (red) and 500-kDa fluorescein-labeled dextran (green) were injected for analyzing kinetics of IgA deposition and visualizing blood vessel wall integrity, respectively. IgA signals were detectable after 2 h in a focal and segmental manner in mice with IgA from gddY mice (Video images S1 and S3). In contrast, mice who received Balb/c IgA did not show clear signals after 2 h.
(MOV)

Video S3 ***In vivo*** **real-time imaging of glomerulus after injection of nephritogenic IgA.** Real-time images of glomeruli at 2 hours after single injection of purified IgA from gddY mice were evaluated by a confocal laser microscopy. Alexa Fluor 633-labeled IgA from gddY mice (red) and 500-kDa fluorescein-labeled dextran (green) were injected for analyzing kinetics of IgA deposition and visualizing blood vessel wall integrity, respectively. IgA signals were detectable after 2 h in a focal and segmental manner in mice with IgA from gddY mice.
(MOV)

Video S4 ***In vivo*** **real-time imaging of glomerulus after injection of IgA from Balb/c mice.** Real-time images of glomeruli at 2 hours after single injection of purified IgA from Balb/c mice were evaluated by a confocal laser microscopy. Alexa Fluor 633-labeled IgA from Balb/c mice (red) and 500-kDa fluorescein-labeled dextran (green) were injected for analyzing kinetics of IgA deposition and visualizing blood vessel wall integrity, respectively. IgA signals were detectable after 2 h in a focal and segmental manner in mice with IgA from gddY mice (Video images S1 and S3). In contrast, mice who received Balb/c IgA did not show clear signals after 2 h.
(MOV)
